# Supplementary material for: Investigating the Prevalence of RNA-Binding Metabolic Enzymes in E. coli
Source: Int J Mol Sci. 2023 Jul 16;24(14):11536. doi: 10.3390/ijms241411536 (PMC10380284; doi:10.3390/ijms241411536)
Supplement: Supplementary file 1 [file ijms-24-11536-s001.zip › ijms-2486990-supplementary.pdf]

Supplementary information

Investigating the Prevalence of RNA-Binding Metabolic Enzymes in *E. coli*

Thomas Klein[1], Franziska Funke[1], Oliver Rossbach[2], Gerhard Lehmann[3], Michael Vockenhuber[4], Jan Medenbach[3], Beatrix Suess[4], Gunter Meister[3] and Patrick Babinger[1,*]

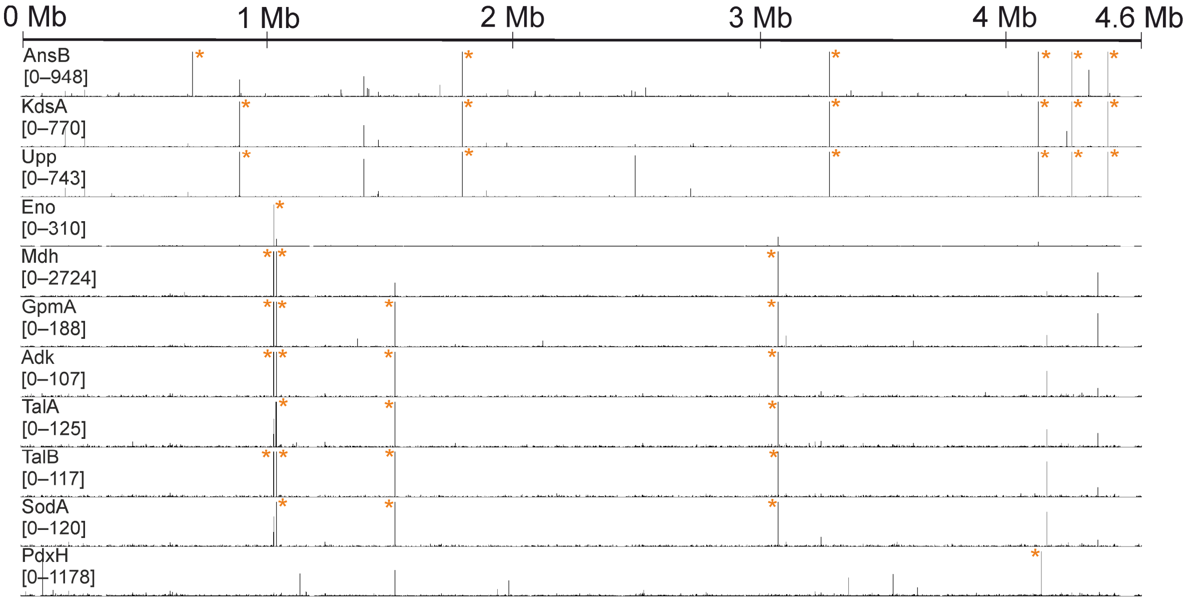

Figure S1. Distribution maps of enzymes that did not produce unique read clusters. The Y-axis is scaled to 2 % of respective total read number for each lane (the numbers under the protein name give this range). Asterisks mark read numbers surpassing the displayed scale. Strong read clustering is co-occuring at same genomic loci between samples, indicating that artifical bias was defining the library selection process. Such artificial bias can be introduced by the method itself, e.g. diverging retention of RNAs during filter binding. In absence of a strong, protein-induced selection pressure, such effects should become more likely to dominate the evolution of the RNA library.

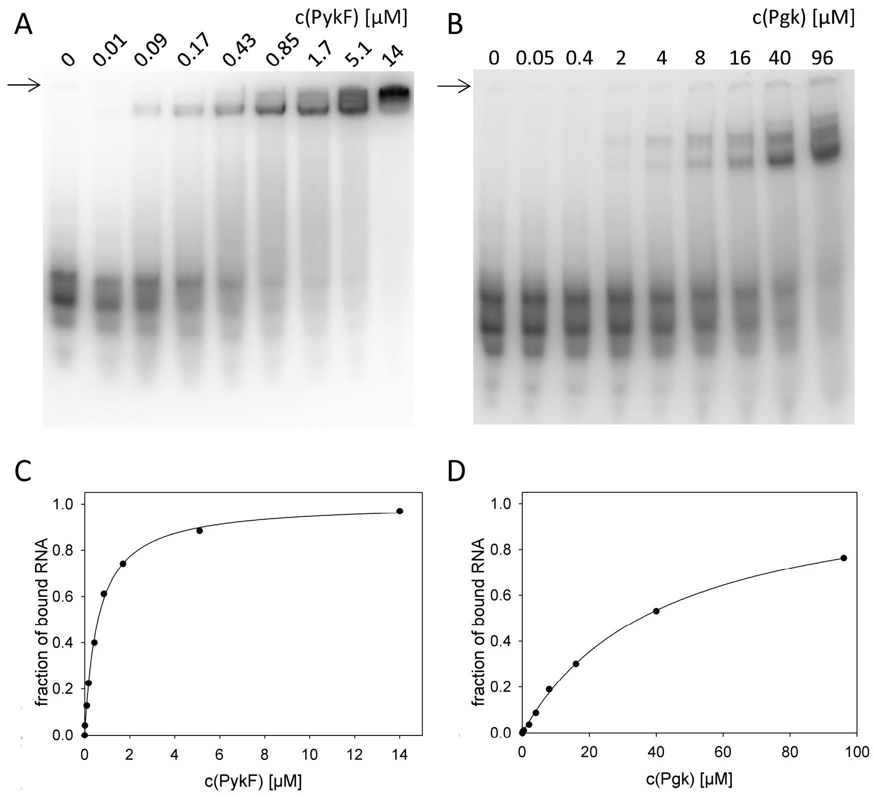

Figure S2. Quantitative EMSA analysis of PykF and Pgk. Different concentrations of (A)PykF or (B) Pgk were incubated with 700 nM radioactively labeled RNA of arbitrary unspecific sequence (see Table S5) and analyzed on native polyacrylamide gels. The protein concentrations are indicated above each lane. Exposure to a phosphor screen enabled visualization of radiolabeled RNA. The two distinct bands of free RNA indicate the formation of a stable secondary structure within the arbitrarily chosen sequence. Arrows indicate the starting point of the gel. (C, D) Band intensities were determined, and the ratio of bound RNA was plotted against protein concentrations in µM. The hyperbolic binding equation was fitted to the data to estimate K_d values: K_d(PykF) = 0.6 µM, K_d(Pgk) = 42 µM.

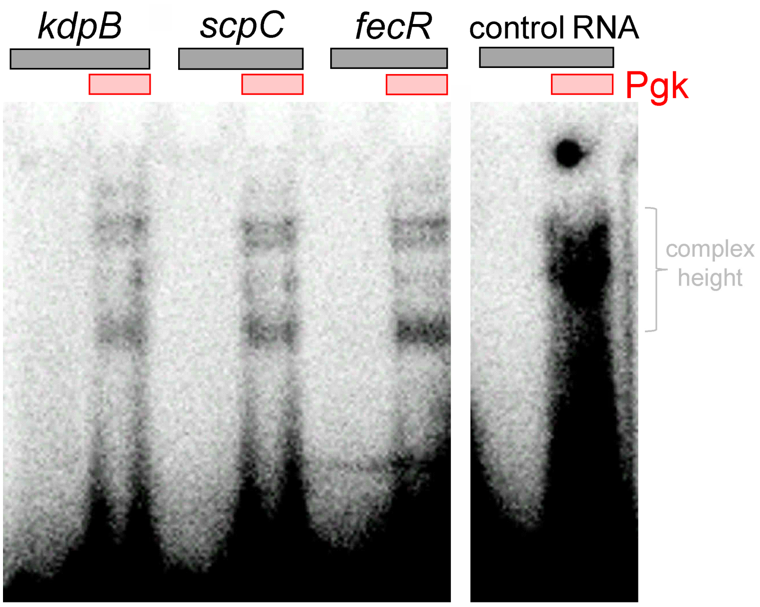

Figure S3. RNA binding of Pgk. EMSA employing 30 µM Pgk (labelled in red) and 7 µM of respective 32P-labeled RNA. Complex shifted band height is indicated. RNA sequences are listed in Table S5.

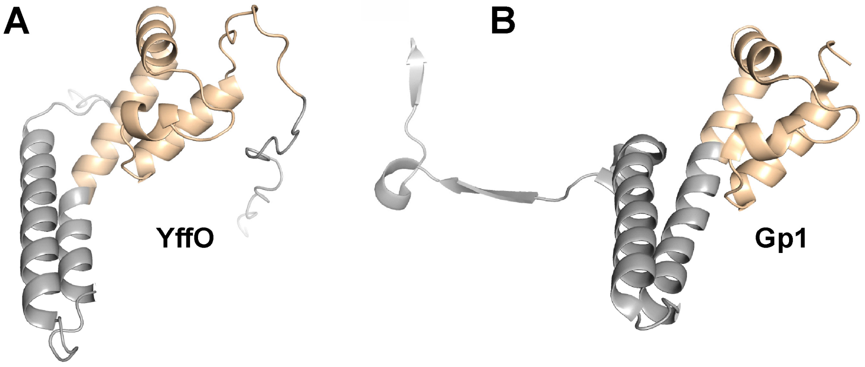

C

```
                                      ____________________________
YffO        1 MEGELIENNGLDIYDTSETPKKRGRPAKYNEKIATQIVLLVSEGYSLRKI     50
                           .....|..|.|||:.|..::|..|..|:|.|.||.|:
Gp1         1 -------------GSHMATEPKAGRPSDYMPEVADDICSLLSSGESLLKV     37

              _______
YffO       51 SMMPGMPSHRQMMRWQLEHMDFREGIAWMSWLWCAEAGRRAVEIIDEVDI    100
              ...||||....:.||..:|.|||:     .:....||  ||..|.:|:
Gp1        38 CKRPGMPDKSTVFRWLAKHEDFRD-----KYAKATEA--RADSIFEEI--     78

YffO      101 NAEDGPKQLRKAEAKAKALL-------AAAKLNSLKHSPFGDDKQ-----    138
               .|.....:..|...|||.|       |.|::|..|   :||...
Gp1        79 -FEIADNAIPDAAEVAKARLRVDTRKWALARMNPRK---YGDKVTNELVG    124

YffO      139 -------------------    138

Gp1       125 KDGGAIQIETSPMSTLFGK    143
```

Figure S4. Features of YffO. (A) AlphaFold prediction (AlphaFoldDB, [109, 110]) of the structure of YffO. Suspected DNA-binding domain colored in beige. (B) Crystal structure of Gp1 (PDB entry: 1HEF, Zhao et al. 2010). DNA-binding domain colored in beige. (C) Global pairwise sequence alignment between YffO and Gp1 (emboss needle [111]). Green bar indicates the AT-hook motif followed by the helix-turn-helix motif for both sequences.

Table S1. Performance of the studied proteins in high throughput studies.

#	#	Kljk#wkurxjksxw#vfuhhqlqj#uhvxow[d#]		
phwderolvp#	hq}|ph#	SWH[# +IF0ydoxh,#	WUDSS## +IF0ydoxh,#	RRSV# +uhsolfdwhv,#
jo|frq|vlv#	S|nI#	0#	51687#	828#
	Sjn#	318:8#	51489#	828#
	JdsD#	0415;:#	51<::#	828#
	Hqr#	031868#	51:59#	828#
	JspD#	031373#	51<98#	828#
shqwrvh# skrvskdwh# sdwkzd|#	WdoD#	0#	51764#	828#
	WdoE#	03139:#	51735#	828#
	UslD#	41356#	61563#	828#
dsrsro|0 vdffkdulghv#	NgvD#	3188:#	613;;#	728#
qxfohrwlgh# phwderolvp#	Xss#	4155<#	51937#	828#
	Dgn#	05169;#	6136:#	828#
	Wk|D#	0#	41:7:#	0#
dplqr#dflg# phwderolvp#	DqvE#	31689#	0#	828#
	SurE#	0#	6136;#	0#
flwulf#dflg#f|foh#	Pgk#	31744#	41;77#	828#
	DfqE#	051633#	41357#	828#
r{lgruhgxfwdvhv#	VrgD#	4134:#	71566#	0#
	TruD#	0#	41::6#	528#
	Sg{K#	0#	51383#	0#

[a] FC-values are the log_2-fold change in protein intensity between crosslinked and non-crosslinked samples, as detected by mass spectrometry. For OOPS, the table lists the number of replicates out of 5 that showed statistically significant FC-values >0, staying in line with original result presentation. TRAPP trialed varying UV dosages, data from highest irradiation is listed. All values are extracted from Urdaneta et al. [32], Shchepachev et al. [34], and Queiroz et al. [33].

Table S2. List of genomic loci where significant read clustering occurred in the MS2 coat protein SELEX experiment.

jhqh#orfxv#	vhqvh2dqwlvhqvh[d]#	(#wrwdo#uhdgv[e]#
gdfE#	dv#	6;1<<#(#
uiiJ#	v#	47178#(#
whvD#	v#	<17<#(#
vvdH#	dv#	;15;#(#
idR#	dv#	9198#(#
dd{#	dv#	7185#(#
hfsF#	v#	7178#(#
hdiJ#	dv#	31;6#(#
pqpD#	dv#	31:7#(#
|klQ#	v#	31:3#(#
puhG#	dv#	3197#(#
|hMO#	dv#	3174#(#
gdp[#	dv#	3174#(#
hqwI#	dv#	3169#(#
|jmL#	dv#	3167#(#
pgwF#	dv#	3164#(#
efvI#	dv#	3164#(#
sssD#	dv#	3159#(#
uwpI#	dv#	3159#(#
jvkD#	dv#	3158#(#
rssD#	v#	3157#(#
|mjU#	dv#	3156#(#
pxuI#	v#	3155#(#
joqH#	dv#	3153#(#
wruV#	dv#	3147#(#
|thE#	dv#	3146#(#
{dqS#	dv#	3145#(#
wru]#	dv#	3145#(#
|egK#	dv#	3144#(#
hejU#	v#	3144#(#
|hhM#	v#	3143#(#
surD#	dv#	313<#(#
|emW#	dv#	313<#(#
sdqI#	dv#	313;#(#
w|qD#	dv#	313:#(#
hpuG#	dv#	313:#(#
qxrN#	dv#	3139#(#
iu|F#	dv#	3139#(#
kusE#	dv#	3139#(#
qxrQ#	dv#	3138#(#
ejd[#	dv#	3138#(#
wusG#	v#	3138#(#
|djJ#	v#	3137#(#
|kiJ2lf#	v#	3137#(#
uleG#	dv#	3136#(#
wrsD#	dv#	3136#(#
|thF#	v#	3136#(#

[a] read orientation relative to orientation of underlying gene
[b] number of reads in this cluster divided by the total number of mapped reads

Table S3. List of genomic loci where significant read clustering occurred in the GapA SELEX experiment.

jhqh#	vhqvh2# dv[d]#	(#wrwdo# uhdgv[e]#	jhqrp lf#frqwh{w[f]#
fp rE#	dv#	31;;#(#	acuggcacag**aaa**c**a**g**uu**gcguagggucgauccccaccgcgaggugc
|lkD#	dv#	31;;#(#	accguuaaacgccaguacagcuucacgcacc**auauu**c**aauu**gcgcuu
|glT#	dv#	319;#(#	accguuggcggc**u**c**auu**g**uu**gcagaacucgaaagugcgcaaagacgugcu
hqwH#	v#	318;#(#	cgcacucauaacgacuacuacuacagcgugcguc
flw[#	v#	317<#(#	gaugccugcaacgucaac**uaaaa**c**ca**c**aaa**gcuugcg
wuxd#	dv#	3179#(#	cacaaccagccggagagcuggauagcagaguugcuggc
p dd[#	dv#	3164#(#	aauaccgaacaccacgaagucg**au**g**auauu**gccgucgguau
freW#	dv#	3164#(#	gucc**aaaa**gc**aa**c**uuuu**cagccugacggcgacucauugccggagcuga
xp sK#	v#	3154#(#	aucgcagagg**uauaaaa**c**a**cgcugucagg
ggsD#	dv#	3153#(#	gguagaccaggaaauggcccgacgcagauccgcc
|ifX#	v#	314;#(#	ucauugcggacuacagcaucacugcgcaaacacgacacg**aa**g**aaaau**ggcggcga
|djH#	v#	314;#(#	uucuuccugggcagcgguggcgaguucucccagcucggcgccgaagagcguaaag
|he]#	v#	3149#(#	ccugccucaugcgcggu**acaa**g**aa**c**auaa**cgauuagccagcg
g{u#	v#	3147#(#	agugcg**aaa**c**uu**c**uuaaaa**cgaugcuac
sdxG#	v#	3146#(#	cc**u**c**auuuau**gcccgucuuauccguuuccgcuuugcccuucacc
kriS#	dv#	3146#(#	uugcugcacccgucgcc**auuu**c**uuuu**gccc
|khv#	v#	3145#(#	aucuuuacgugccugggcgcuguucgc**auu**c**u**c**uuuu**ggcgcuucgucg
|diQ#	dv#	3145#(#	uugcugaacaagagg**a**g**aaaaa**gcccauaaaggcgcg
duiE#	v#	3144#(#	uucc**uu**c**uuuuuauu**cccgaccgcugcgc

[a] RNA orientation with respect to the gene (irrespective of the gene being located at + or – strand).
[b] Read numbers listed as percentage of global number of mapped reads. The table shows all hits in the GapA experiment with read numbers > 0.1%.
[c] Transcript of genomic sequence colocalizing with apex of read cluster coverage. AU-rich stretches are marked in bold and underlined.

Table S4. List of genomic loci where significant read clustering occurred in the ThyA SELEX experiment.

Jhqh#orfxv#	vhqvh2dqwlvhqvh[d]#	(#wrwdo#uhdgv[e]#
usvF#	dv#	6:193#(#
|kg\#	v#	5<193#(#
up G#	dv#	4:189#(#
xjsT#	v#	<167#(#
|tkG#	dv#	31<<#(#
|kfE#	v#	31:6#(#
|mhI#	v#	3187#(#
ihfE#	v#	3186#(#
dfhD#	dv#	316:#(#
|mkS#	dv#	314:#(#
kfs#	v#	3148#(#
qdjH#	dv#	3147#(#
k|sI#	v#	3145#(#
|ejO#	v#	313<#(#
|mjU#	v#	313:#(#
sssD#	v#	313:#(#
sxwD#	v#	3136#(#
p hwH#	dv#	3136#(#
sioF#	v#	3135#(#
idK#	v#	3135#(#
xyuE#	v#	3135#(#
jss#	dv#	3135#(#
|mfR#	dv#	3134#(#

[a] RNA orientation with respect to the gene (irrespective of the gene being located at + or – strand).
[b] Read numbers listed as percentage of global number of mapped reads

Table S5. Sequences of RNA fragments and DNA oligonucleotides used in this study.

h{shulphqw#	UQD#2#GQD#	JhqEdqn#hqwu|#	UQD#2#GQD#vhtxhqfh#+8*#A#6*,#
#	#	#	
UQD#iudjphqw#xvhg#iru#HPVDv			
PV5#frdw#surwhlq# +Ljxuh#6,#	uiiJ#p UQD#iudjphqw#	QFb333<46=6<:5:4<06<:5:87#	GCACGCGUAUUCACUGAGCAUCAGCCAGAC UGUGU
	frpshwlwru#UQD#	0#	GGGUUCUAGAGAGGUGAGCUUGGCAACCUC UGAUGUAGGU
#	#	#	
J oxwdp dwh#nlqdvh# +Ljxuh#:,#	wnwD#dvUQD#iudjphqw#	QFb333<46=63;38<8063;3953#	UUUAGCGAUGAACUCUUUCGCUUUAG
	frqwuro#UQD04/# h{fhvv#frqwuro#UQD04#	0#	AUCUACCGGCACGCGAUCGCCGGUCGU
	frqwuro#UQD05#	0#	GAGCCACCGUCAGAUGAUGCGCUGGCA
	h{fhvv#frqwuro#UQD05#	0#	AUAGUUUGCGCAAGAUCAGCAUGAUGC
#	#	#	
T xlqrqh# r{lgruhgxfwdvh# +Ljxuh#;,#	|iiR#p UQD#iudjphqw#	QFb333<46=5895644058956 6:#	GGAGCAUAUGGACUUCAGGGAAGGGAU
	frqwuro#UQD#+Ljxuh#;E,#	0#	CCAGCGCGCAGCAGAGUUGCUGCGCUG
#	frqwuro#UQD#+Ljxuh#;F,#	0#	AUGUCAGCACGCAGAGUGUGCAGCGGU
#	#	#	
S|uxydwh#nlqdvh#2# skrvskrjo|fhudwh# nlqdvh#+Ljxuh#V5,#	frqwuro#UQD#	0#	GGGUUCUAGAGAGGUGAGCUUGGCAACCCU GAUGUAGGU
#	#	#	
Skrvskrjo|fhudwh# nlqdvh#+Ljxuh#V6,#	ngsE#UQD#	QFb333<46=#:59;6:0:59;89##	GCCGCGCUAAACAGCGCAUU
	vfsF#UQD#	QFb333<46=#6397<7:06397<99##	AUGAGGCCAAAAAGCCGUAU
	lhfU#UQD#	QFb333<46=#784:3:40784:3<3##	CCCGCGCAAAAACGCAUCGU
	frqwuro#UQD#	0#	AGGACAAAAACAA
#	#	#	
GQD#rojrqxfohrwlghv#xvhg#lq#VHOH[			
wuxqfdwhg#dgdswhu# sulphuv#	W:0I#	#	GTATAATACGACTCACTATAGGGACACTCT TTCCCTACACGAC [a]
	1:0uhy#	#	GTGACTGGAGTTCAGACGTG
dgdswhu#sulphuv# iru#uhvwrudwlrq#	I8bdgdswhubuhvwruh#	#	AATGATACGGCGACCACCGAGATCTACACT AAGATTAACACTCTTTCCCTACACGA
	L:bdgdswhubuhvwruh#	#	CAAGCAGAAGACGGCATACGAGATTTCTGA ATGTGACTGGAGTTCAAGACGTG

[a] the underlined sequence is the T7 promoter sequence plus the first three transcribed bases ("GGG"). The following 20 adapter bases are added as a constant sequence to each transcript.
